# Manipulation of Mitochondrial Plasticity Changes the Metabolic Competition Between “Foe” and “Friend” During Tumor Malignant Transformation

**DOI:** 10.3389/fonc.2020.01692

**Published:** 2020-08-21

**Authors:** Hui Tian, Baofu Zhang, Liantao Li, Gang Wang, Huizhong Li, JunNian Zheng

**Affiliations:** ^1^Cancer Institute, Xuzhou Medical University, Xuzhou, China; ^2^Center of Clinical Oncology, Affiliated Hospital of Xuzhou Medical University, Xuzhou, China; ^3^Jiangsu Center for the Collaboration and Innovation of Cancer Biotherapy, Cancer Institute, Xuzhou Medical University, Xuzhou, China

**Keywords:** mitochondrial plasticity, tumor metastasis, therapeutic resistance, T cells exhaustion, memory T cells

## Abstract

Mitochondria as the cellular energy powerhouses provide a common site for multiple metabolic reactions in order to cover energy and biomolecule demands, thus integrating the diverse metabolic pathways to endow cells with metabolic adaptation. Mitochondrial plasticity is normally regulated by mitochondrial dynamics, mitochondrial metabolism and mitochondrial biogenesis. Given that tumor cells and T cells share the metabolic similarities of survival, proliferation, expansion as well as effector function, manipulation of mitochondrial plasticity would change the metabolic competition between “foe” and “friend” during tumor malignant progression. On the one hand, for “foe” tumor cells, mitochondrial plasticity provides the enhancement of tumor metastasis and the development of resistance to‘ diverse antitumor drugs. On the other hand, for “friend” T cells, mitochondrial plasticity promotes the generation of long-term memory T (T_M_) cells and alleviates the exhaustion of tumor-infiltrating lymphocytes (TILs). Therefore, downregulation of mitochondrial plasticity of tumor cells through engineering tumor-targeting nanoparticles may effectively potentiate metabolic vulnerability and re-sensitize tumor to relevant therapeutic treatment. On the contrary, upregulation of mitochondrial plasticity of T cells through optimizing adoptive cellular immunotherapy (ACI) or chimeric antigen receptor (CAR)-T cell therapy would provide T cells with the robust metabolic fitness and the persistent immune function, thus blocking tumor metastasis and reoccurrence.

## Introduction

Mitochondria as the dynamic subcellular organelles are capable of rapid sensing energy availability and are involved in production of precursors for the biosynthesis of amino acids, lipids, and nucleotides to meet cellular requirement for rapid proliferation (1–3). In addition, mitochondria also sense the extracellular adverse stimulation and coordinate the intrinsic apoptotic signaling pathways to control cell death (4, 5). Actually, mitochondria are regarded as the hub of numerous signal pathways including tricarboxylic acid (TCA) cycle, oxidative phosphorylation (OXPHOS) and fatty acid oxidation (FAO), which endows cells with considerable metabolic flexibility to adapt to the various stresses and develop the therapeutic resistance.

In this review, we discuss mitochondrial plasticity as a “double-edged sword” during tumor malignant transformation because that “foe” tumor cells depend on it to develop tumor metastasis, relapse as well as therapeutic resistance (6–8), whereas “friend” T cells require it to exert the robust and persistent anti-tumor immune response (9–11). Given that mitochondrial plasticity is tightly linked to mitochondrial dynamics, mitochondrial metabolism and mitochondrial biogenesis, recent reports have highlighted the importance of mitochondrial plasticity on the metabolic competition between tumor and T cells. Therefore, the development of respective mitochondria-targeted antitumor strategies could not only significantly re-sensitize tumor cells to therapeutic treatment but also dramatically improve T cells metabolic fitness to maximize their efficacy of related immune therapy.

## Mitochondrial Plasticity and Tumor Malignant Progression

Mitochondria have attracted considerable attention as targets for the development of novel antitumor agents because that they are not only linked to tumor onset, progression and malignant transformation but also to tumor survival in adverse stresses such as nutrient deprivation and therapeutic drugs induced DNA damage. The metabolic plasticity is primarily regulated by (1) mitochondrial dynamics, (2) mitochondrial metabolism, and (3) mitochondrial biogenesis, which always contributes to tumor malignant transformation and therapeutic resistance.

### Mitochondrial Dynamics Is Involved in Development of Tumor Therapeutic Resistance

Mitochondria as the dynamic organelles switch their morphology between mitochondrial fission and fusion according to cellular bioenergetic requirements (12, 13). Mitochondrial architecture exhibits the constant shift between fusion and fission, which is closely controlled by a number of key proteins such as dynamin-related protein 1 (Drp1), optic atrophy 1 (OPA1) and mitofusin 1/2 (MFN 1/2) (14–16). The alteration of mitochondrial morphology is important in the maintenance of mitochondrial metabolism for cellular energy supply and survival.

Some previous studies suggest that mitochondrial morphology is related to cellular metabolic pattern. Mitochondrial fusion contributes to the tight association of electron transport chain (ETC) complex, which prefers to OXPHOS and fatty acid oxidation (FAO) (17, 18). On the other hand, mitochondrial fission due to the loose mitochondrial cristae favors aerobic glycolysis (19). Therefore, reduced OXPHOS and enhanced glycolysis are correlated with the fragmented mitochondria. In other words, dysfunctional mitochondria switch cellular metabolism from mitochondria-induced OXPHOS to aerobic glycolysis.

The involvement of mitochondrial dynamics in tumor progression is documented in multiple studies (20, 21). At the beginning of tumorigenesis, tumor cells always exhibit the increased fission and/or decreased fusion, which favors aerobic glycolysis to cover tumor cells request for rapid proliferation and expansion. However, the emergence of resistant populations is associated with mitochondrial fusion (22, 23). Facing therapeutic treatment, mitochondrial fusion provides the cellular protection from DNA damage-mediated apoptosis. Mitochondrial elongation due to mitochondrial protein rearrangement is helpful to resist cisplatin-induced treatment in neuroblastoma cells while silencing of MFN1 inhibits mitochondrial fusion but increases cisplatin sensitivity (24). Besides, OPA1 overexpression displays more resistance to cisplatin treatment in lung adenocarcinoma cells (25), whereas OPA1 downregulation promotes the release of cytochrome c, thus inducing apoptosis and attenuating the cisplatin resistance (26). These studies suggest that a high level of mitochondrial fusion always exists in resistant tumor cells, while the dominant mitochondrial fission is easier to sensitize tumor to chemotherapy drugs.

Considering that metabolic stressors such as nutrient deprivation due to tumor cells rapid expansion, chemotherapy and radiotherapy act as a selection pressure to tumor cells, alteration of the mitochondrial architecture and dynamics enable the metabolic adaptation and evasion of cell death programs to ultimately support tumor proliferation, migration and drug resistance.

### Mitochondrial Metabolism Is Implicated in Tumor Malignant Transformation

Mitochondria regulate cellular bioenergetic and biosynthetic processes to be involved in several central signaling pathways such as tumor proliferation, metastasis and self-renewal (6). Although “Warburg Effect” pointed out that the aerobic glycolysis dominated in tumor metabolism and was associated with tumor rapid proliferation and development (27), the high rate of glycolytic metabolism in tumors failed to be attributed to mitochondrial dysfunction (28, 29).

The several highly proliferative tumor cell lines did not been found the defects in mitochondrial oxidative metabolism (30). On the contrary, transformed mesenchymal stem cells switched their metabolic dependency to OXPHOS (31). In addition, oxidative metabolism may recycle the excess glycolysis production lactate critical for tumorigenesis. Mitochondrial OXPHOS function, therefore, is advantageous in highly glycolytic tumor cells (32–34). Furthermore, some oncogenes known to promote the use of the glycolytic pathway can also upregulate genes important for mitochondrial OXPHOS metabolism. For example, mitochondrial protein signal transducer and activator of transcription 3 (STAT3) potentiated ETC activity, leading to Ras-dependent malignant transformation (35). Fogal et al. also reported that loss of mitochondrial protein p32, known as an important executor required for mitochondrial function and OXPHOS, suppressed mitochondrial protein translation and negatively affected a number of electron transport complexes function, thus resulting in the repression of tumor proliferation and metastasis (36). p32 induced mitochondrial bioenergetics are now regarded to be positively related to the tumorigenic and metastatic potential in tumor cells.

Actually, some tumor cells harbor a highly flexible metabolic phenotype, whereby they could use both glycolysis and the byproducts from glycolysis by OXPHOS to increase their “cellular fitness” and improve their survival under harsh conditions. The potential role of metabolic plasticity in tumor metastasis and therapy resistance has been paid more attention (37, 38). A hybrid metabolism could maintain low reactive oxygen species (ROS) levels which induce a moderate stress response and the appearance of mutations that further stimulate tumorigenesis and metastasis (39, 40). Therefore, dual inhibition of glycolysis by 2-Deoxy-d-glucose (2-DG) and OXPHOS by metformin has been shown to effectively block tumor malignant transformation (41).

Taken together, these studies suggested that mitochondrial OXPHOS was indispensable for tumor progression. The oncogene-driven glycolytic metabolism should be balanced at least in part by concomitant changes to mitochondria, whereas shifting the balance too far from OXPHOS was detrimental for tumor malignant transformation. In this regard, mitochondrial metabolism enables tumor cells to cooperate with glycolysis and modulate their metabolic pattern according to tumor progression demands, thus providing metabolic flexibility to be involved in tumor metastasis and therapeutic resistance.

### Mitochondrial Biogenesis Favors the Maintenance of Tumor Stemness

Mitochondrial biogenesis is essential for the renewal of damaged mitochondria, as well as the maintenance of the bioenergetic and biosynthetic demands of the cellular development (42, 43). Recently, mitochondrial biogenesis has been paid more attention due to the tight link to cancer stem cells (CSCs). CSCs are a population of cells with stem cell-like properties that are considered to be the root cause of tumor heterogeneity and are responsible for metastatic dissemination and therapeutic resistance (44, 45). As such, targeting CSCs may be a useful strategy to improve the effectiveness of classical anticancer therapies. The current studies on the metabolic features of CSCs primarily focus on the biochemical energy pathways involved in CSCs maintenance and propagation (46, 47).

Mitochondrial biogenesis has been reported to deeply influence the properties of CSCs, as well as their transition from a quiescent to a proliferative state. Some evidence shows that CSCs are characterized by the increased mitochondrial mass and the elevated mitochondrial biogenesis, whereby CSCs increase their self-renewal capacity in diverse cancer types independent of their glycolytic- or OXPHOS-dependent metabolic phenotype (48–50). In this scenario, mitochondrial biogenesis accompanied with increased membrane potential, higher generation of mitochondria-derived ROS, and enhanced mitochondrial mass as well as oxygen consumption, has recently emerged as a key feature of CSCs when compared with the differentiated cells in the tumor bulk (51–53). Therefore, mitochondrial biogenesis is not only a pivotal player but also a regulatory instigator of tumor stemness.

The metastatic potential of CSCs has been reported to be related to the activity of peroxisome proliferator-activated receptor gamma coactivator 1 alpha (PPARGC1A, also known as PGC1α). PGC1α as a transcription coactivator regulates mitochondrial biogenesis and is involved in energy metabolism. Besides, PGC1α has been shown to couple OXPHOS to enhance migratory and invasive capability of CSCs, as revealed by using human invasive breast tumor samples (47, 54). Of note, PGC1α overexpression had been identified in circulating tumor cells as well as in breast CSCs while its inhibition reduced stemness properties. These studies suggested that PGC1α-mediated mitochondrial biogenesis played an important role on tumor stemness maintenance via activation of mitochondrial metabolism. In this regard, targeting mitochondrial biogenesis has been demonstrated to improve the particular therapeutic efficacy for cancers especially CSCs with malignant phenotypes. Therefore, potentiation of expression of PGC1α enhanced mitochondrial biogenesis to maintain CSCs stemness while knockdown of PGC1α impaired breast CSCs survival and propagation, which suggested that PGC1α-mediated mitochondrial biogenesis was necessary for the maintenance of CSCs stemness.

**FIGURE 1:**
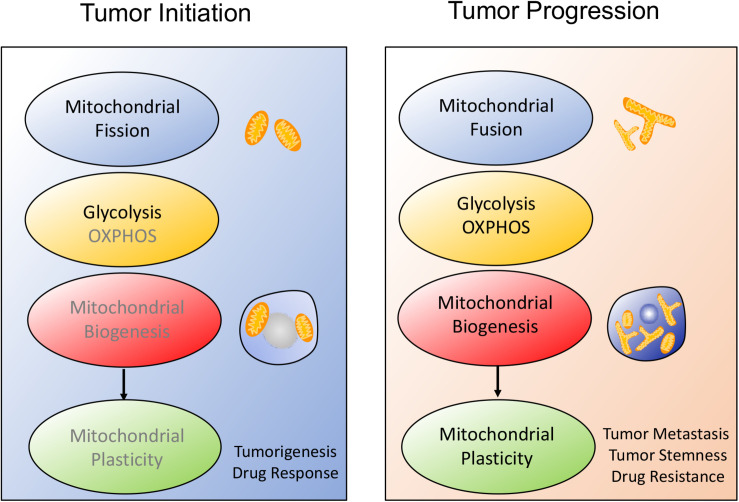
Schematic overview of the alteration of mitochondrial plasticity during tumor development. The mitochondrial plasticity including mitochondrial dynamics, mitochondrial metabolism and mitochondrial biogenesis, is closely related to the stages of tumor development. (1) During tumor onset, mitochondrial fission is always consistent with increased aerobic glycolysis and decreased mitochondrial biogenesis, which covers tumor cells request for rapid proliferation and sensitizes tumor to therapeutic drugs. (2) Once tumor starts malignant transformation, mitochondrial morphology moves toward fusion, possibly protecting tumor from apoptosis. At the same time, tumor prefers to OXPHOS metabolism and improves mitochondrial biogenesis, thus facilitating tumor metastasis and therapeutic resistance.

Interestingly, some investigations reported that mitochondrial biogenesis was also coupled to mitochondrial dynamics and mitochondrial metabolism, which further affected tumor metastasis, migration and stemness. Actually, tumor engages the distinctive metabolic reprogramming to drive the different stage of tumor development, as shown in Figure 1. During tumor initiation, mitochondrial fission blocks mitochondrial function and favors aerobic glycolysis to support tumor initiation and proliferation. Aerobic glycolysis dominates cellular metabolism while OXPHOS is temporarily depressed due to the several signaling pathways activation such as PI3K/AKT, RAS/RAF/MAPK, hypoxia-inducible factor-1α (HIF1α) (55–57). In addition, mitochondrial biogenesis fails to display the significant enhancement. However, once tumor undergoes more complicated malignant transformation, mitochondrial biogenesis enhancement is accompanied with mitochondrial fusion and mitochondrial OXPHOS, which finally improves mitochondrial plasticity and endows tumor with adaptability, migration, stemness and therapeutic resistance.

## Mitochondrial Plasticity and T cells Immune Function

Metabolism fuels T cells all biological programs, ranging from development, proliferation, differentiation, and the effector functions (58–60). During this process, metabolic reprogramming provides T cells for the distinct energetic and functional needs depending on their state of activation. During the quiescent state, naïve T (T_N_) cells primarily rely on OXPHOS as their primary pathway of ATP production. Upon antigen stimulation, T cells undergo a proliferation phase of cell mass accumulation followed by cell cycle entry, clonal expansion, differentiation into effector T (T_EFF_) cells that prefer glycolysis to OXPHOS for biomass accumulation and effector functions. However, after pathogen clearance or tumor suppression, effector T cells shrink and undergo apoptosis, leaving behind a small population of long-lived memory T (T_M_) cells characterized by a different metabolism, with increased reliance on FAO to fuel OXPHOS, which can respond vigorously upon antigen rechallenge.

These studies suggest that T cells metabolism is not static but rather a dynamic process to meet bioenergetic demands of T cells at any point in time. Here, we discuss the potential methods targeting T cells mitochondrial plasticity effectively improve their immunotherapy efficiency to dampen tumor malignant progression and reoccurrence.

### Mitochondrial Plasticity Is Associated With T Cells Fate

**FIGURE 2:**
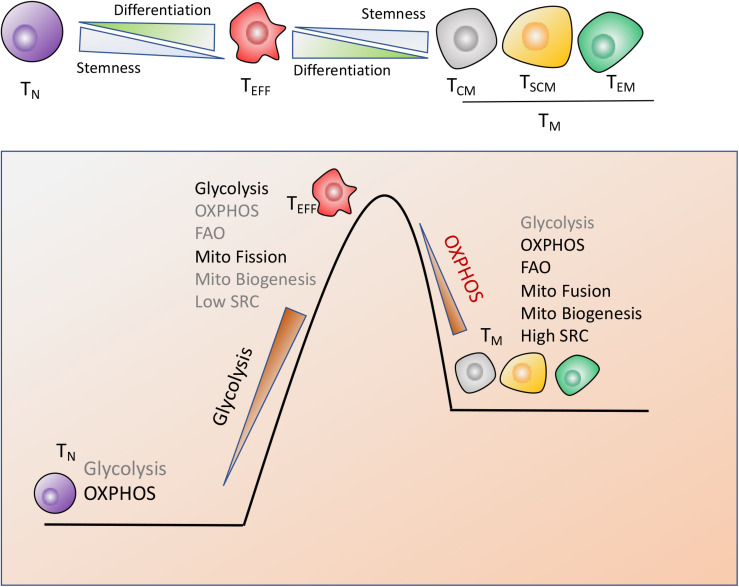
Metabolic reprogramming drives T cells differentiation. CD8^+^ T cells are composed by naïve (T_N_), stem cell memory (T_SCM_), central memory (T_CM_), effector memory (T_EM_) and terminally differentiated effector cells (T_EFF_), which display the distinct metabolic profiles to perform their respective functions. (1) T_N_ cells maturation mainly rely on OXPHOS. (2) Terminally differentiated T_EFF_ cells are marked by the engagement of aerobic glycolysis, accompanied by suppression of mitochondrial biogenesis and increase of mitochondrial fission, which endows T cells with the rapid and robust effector function. (3) The long-lived T_M_ cells are characterized by enhanced mitochondria-mediated OXPHOS and FAO, dominant mitochondrial fusion and increased mitochondrial biogenesis, which might timely trigger immune response after antigen rechallenge.

Recent works in this field indicated that the modulation of T cells metabolism was linked to T cells differentiation and anti-cancer activity (9, 61, 62). CD8+ T cells are composed by naïve T (T_N_) cells, stem cell memory T (T_SCM_) cells, central memory T (T_CM_) cells, effector memory T (T_EM_) cells and terminally differentiated effector T (T_EFF_) cells. Notably, T cells antitumor capacity was inversely proportional to T cells differentiation. In other words, fully-differentiated T_EFF_was found to be less effective in controlling tumor growth than less-differentiated T_M_ cell subsets such as T_SCM_ or T_CM_ cells with self-renewal potential and increased anti-tumor immunity, as shown in Figure 2.

Naiïve T cells maturation and existence mainly rely on OXPHOS while terminal T_EFF_ cells preferentially utilize glycolysis, accompanied with suppression of mitochondrial biogenesis and increase of mitochondrial fission, which equips T_EFF_ with the robust effector function but compromises T cells long-term function (58). However, the long-lived T_M_ cells are characterized by enhanced mitochondria-mediated OXPHOS and FAO, decreased glycolysis, dominant mitochondrial fusion and enhanced mitochondrial biogenesis, which provides T_M_ cells with self-renewal potential to trigger the rapid and effective immune response once antigen rechallenge (63, 64). In addition, T_M_ cells possess the increased mitochondrial mass, which contributes to the greater mitochondrial spare respiratory capacity (SRC) relative to T_N_ or T_EFF_ populations (64, 65). SRC is regarded as the maximal respiratory potential allowing for rapid mitochondrial ATP production upon T-cell receptor (TCR) engagement, which thus confers a bioenergetic advantage on T_M_ cells upon secondary exposure to antigen (Figure 2).

Consequently, T cells mitochondrial plasticity are collectively determined by mitochondrial fusion, mitochondrial-mediated OXPHOS and FAO, SRC as well as mitochondrial biogenesis, which effectively decreases T cells differentiation and promotes the long-lived T_M_ cells generation. As such, metabolic reprogramming with enhanced mitochondrial plasticity promotes T cells to produce the persistent and robust energy for rapid response to tumor reoccurrence.

### Mitochondrial Plasticity Prevents T Cells Exhaustion

As T cells differentiate during an immune response, they move from nutrient-replete lymphoid organs to nutrient-deficient tumor microenvironment (TME). Restrictive environment forces T cells to perform the metabolic reprogramming for adaptation to the adverse stresses and exert their necessary functions. Metabolic insufficiency may be a fundamental mechanism by which environmental context dampens tumor-infiltrating lymphocytes (TILs) effector function and potentially induces their tolerance and anergy (66, 67). Therefore, manipulating mitochondrial plasticity is an important consideration for TILs, which ensures the maintenance of TILs proliferation and/or effector function.

Programmed cell death protein 1 (PD-1) signals were reported to inhibit the expression of key mitochondrial biogenesis regulator PGC1α (68). Overexpression of PGC1α reversely corrected metabolic alterations in developing exhausted T cells to improve the effector function of these hyporesponsive T cells. Scharping et al. also reported that loss of mitochondrial function in TILs was attributed to a defect in PGC1α-programmed mitochondrial biogenesis, however, independent of PD-1 blockade (69). These studies confirmed that metabolic reprogramming of T cells through enhancement of PGC1α expression effectively rescued mitochondrial function and reinvigorated T cells superior antitumor responses characterized by cytokine production and tumor control.

In addition to mitochondrial biogenesis, mitochondrial plasticity related mitochondrial fusion, mitochondrial metabolism such as OXPHOS and FAO, and mitochondrial reserved capacity SRC are all associated with regulation of TILs exhaustion (70–72). Notably, mitochondrial biogenesis and metabolic reprogramming were not the independent processes. Some studies suggested that potentiation of mitochondrial biogenesis was prone to catabolic metabolism and T_M_ cell formation, thus retarding T cells exhaustion (73, 74). For example, memory CD8+ T cells with low differentiation level can provide long-term protection against tumors, which depends on their enhanced proliferative capacity, self-renewal and unique metabolic rewiring to sustain cellular fitness. On the other hand, overexpression of PGC1α improved the proportion of the central memory T cell in TILs, which mediated more persistent and robust recall responses to antigens.

**FIGURE 3:**
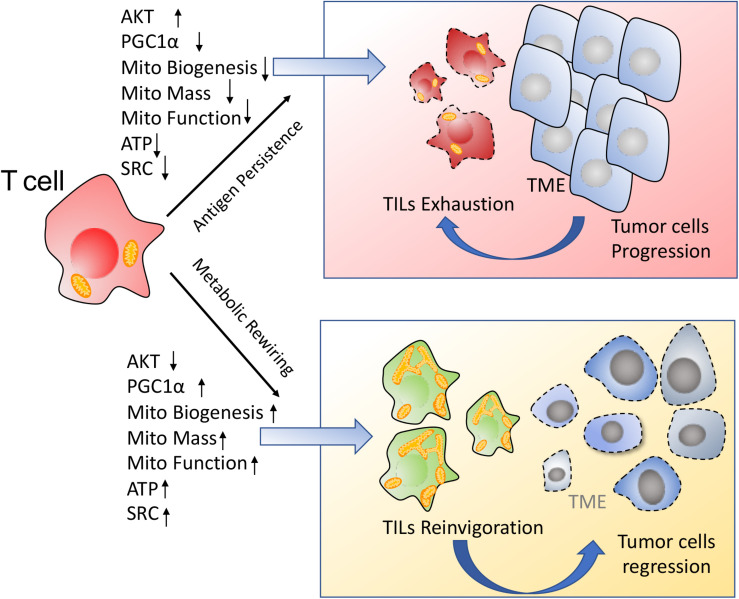
Enhancement of mitochondrial plasticity prevents TILs exhaustion. (1) In response to the chronic tumor antigen stimulation, TILs display the metabolic insufficiency, characterized by a persistent loss of mitochondrial biogenesis and mitochondrial function. These metabolically insufficient TILs exhibit an exhausted phenotype especially under immunosuppressive TME. (2) Metabolic rewiring through enhancement of mitochondrial plasticity reinvigorates TILs to exert the robust immune function for tumor repression.

Together, the chronic activation associated with the anti-tumor response represses TILs mitochondrial plasticity including decreased PGC1α mediated mitochondrial biogenesis and suppression of mitochondrial metabolism, which further drives T cells hyporesponsiveness. On the contrary, improvement of mitochondrial plasticity such as enhancement of mitochondrial biogenesis via PGC1α, augment of mitochondrial metabolism including OXPHOS and FAO, ATP production, SRC as well as regulation of mitochondrial dynamics, are proposed to retard TILs exhaustion and reinvigorate T cells to fulfill durable antitumor immune function, as shown in Figure 3.

## Manipulation of Mitochondrial Plasticity Changes the Metabolic Competition Between Tumor and T cells

**FIGURE 4:**
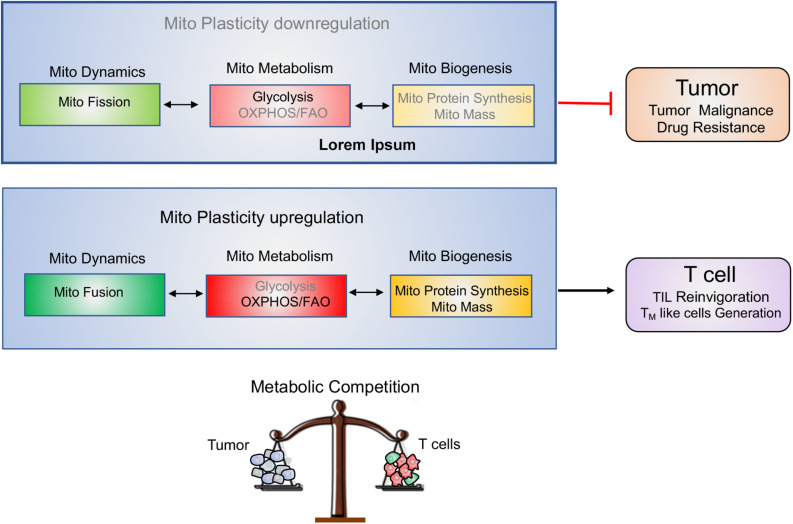
Manipulating mitochondrial plasticity changes the metabolic competition between tumor and T cells. Mitochondrial dynamics, mitochondrial metabolism and mitochondrial biogenesis profoundly affect mitochondrial plasticity. (1) Downregulation of mitochondrial plasticity through promotion of mitochondrial fission, suppression of mitochondrial OXPHOS and reduction of mitochondrial biogenesis effectively dampens tumors malignancy and drug resistance. (2) Upregulation of mitochondrial plasticity through promotion of mitochondrial fusion, enhancement of mitochondrial OXPHOS and increase of mitochondrial mass is responsible for TILs reinvigoration and T_M_-like cells generation. (3) Manipulating mitochondrial plasticity would change the metabolic competition between “foe” tumor and “friend” T cells.

Tumor and T cells share the similar metabolic reprogramming to promote their growth, survival, proliferation, and long-term maintenance (75–77). The different strategies targeting mitochondrial plasticity including mitochondrial dynamics, mitochondrial metabolism as well as mitochondrial biogenesis should be executed on tumor and T cells, respectively. For “foe” tumor cells, downregulation of mitochondrial plasticity not only represses tumor malignant progression but also re-sensitizes tumor to the conventional therapeutic treatments. On the other side, for “friend” T cells, upregulation of mitochondrial plasticity effectively reinvigorates T cells with persistent and robust immune function and promotes T_M_ like cells generation. In other words, manipulation of mitochondrial plasticity changes the metabolic competition between “foe” tumor and “friend” T cells, as shown in Figure 4.

### Suppression of Mitochondrial Plasticity Represses Tumor Malignant Transformation and Therapeutic Resistance

Malignant tumors harbor heterogeneous populations of cells in various states of proliferation and differentiation (78, 79). CSCs characterized by the enhanced mitochondrial adaptations are primarily responsible for tumor metastasis, relapse as well as therapeutic resistance. Some studies further pointed out that multimodal therapies targeting mitochondria called the “Achilles heel” of CSCs would constitute the promising therapeutic strategies (46, 80, 81). These stubborn tumors were armed with the high mitochondrial plasticity like increased OXPHOS and FAO, enhanced mitochondrial biogenesis as well as distinct mitochondrial morphology, which was subsequently involved in the maintenance of tumor stemness, promotion of tumor metastasis and resistance of therapeutic drugs (81–83).

Firstly, OXPHOS-dependent energetic status is partially responsible for resistance to some tumor ablation methods including oxidative stress, radiation, and chemotherapeutics in many hematological and solid tumors (84, 85). Drugs targeting mitochondrial metabolism have been recognized as a potential avenue to suppress tumor progression. Low doses of respiratory complex I (CI) inhibitors such as rotenone, piericidin A or capsaicin were selectively toxic to tumor cells especially under glucose depletion (86). These cells death mechanism was strongly dependent on the inherent mitochondrial function. Interestingly, tumor cells with higher glycolysis were more susceptible to CI inhibitors. In addition, drugs stimulating OXPHOS such as the cAMP-dependent protein kinase (PKA) activator forskolin strongly enhanced the cytotoxic effect of CI inhibitors. In other words, stimulation of OXPHOS in presence of low glucose in the microenvironment would make glycolytic tumor cells more sensitive to mitochondrial respiratory chain inhibitors. Besides, Lee et al. reported that chronic myeloid leukemia (CML) was founded to rely on mitochondrial energetic metabolism (87). Consequently, combination of the mitochondrial protein translation inhibitor tigecycline with tyrosine kinase blocker imatinib was found to selectively eradicate CML stem cells. Actually, some conventional chemotherapy drugs such as 5-fluorouracil, cytarabine, tyrosine kinase inhibitors (TKi), BRAF inhibitors (BRAFi) and MAPK inhibitors (MAPKi), promoted mitochondrial OXPHOS activity to easily induce resistant clone expansion, whereas some drugs such as anthracyclines, etoposide, sorafenib, taxol, and staurosporine, significantly depressed mitochondrial function, thus facilitating the clearance of these resistant cancer cells depending on oxidative metabolism (88). Of course, the origin of the therapeutic resistance due to either OXPHOS-dependency or Warburg-related features still remains controversial.

Secondly, mitochondrial biogenesis as the indispensable regulation factor of mitochondrial plasticity has been reported to allow tumor rapid adaption to the limited nutrient availability and be associated with tumor malignant transformation. Tumors always increase their mitochondrial biogenesis and bioenergetics to rescue them from energy scarcity and promote tumors invasion as well as metastasis. Actually, PGC1α, the main regulator of mitochondrial biogenesis has been found to be overexpressed in several cancer types especially CSCs subsets and contribute to tumor malignant progression (89, 90). Although targeting PGC1α could help to overcome therapeutic resistance, there are no specific inhibitors of PGC1α available to date. Therefore, indirect inhibition through upstream modulators of PGC1α to regulate mitochondrial biogenesis may be a viable measurement (91–94). In fact, MAPK inhibitor-resistant melanoma cells were sensitized to therapy when treated with mTORC inhibitors, which decreased PGC1α expression and reversed metabolic changes. Moreover, some BRAF-mutated melanoma cells are intrinsically resistant to MAPKi. Targeting mitochondrial biogenesis with a mitochondria-targeted, small-molecule HSP90 inhibitor (Gamitrinib) eradicated intrinsically resistant cells and augmented the efficacy of MAPKi through inducing mitochondrial dysfunction and inhibiting tumor bioenergetics (95). These above data suggested that targeting mitochondrial biogenesis as well as aberrant tumor bioenergetics provides a rationale-based combinatorial strategy to improve the efficacy of the conventional therapeutic drugs.

**FIGURE 5:**
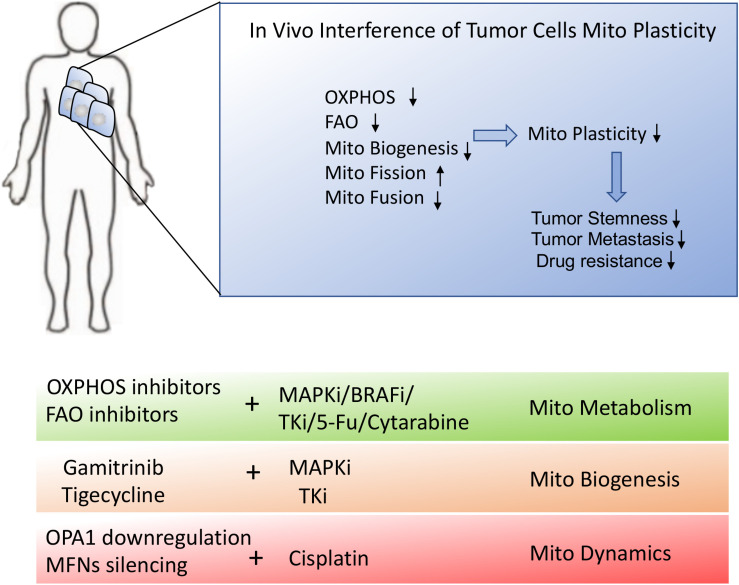
Schematic presentation of *in vivo* interference of tumor metabolic reprogramming. *In vivo* interference of tumor mitochondrial plasticity through suppression of OXPHOS, mitochondrial fusion and mitochondrial biogenesis may dramatically inhibit tumor malignant progression and therapeutic resistance. The combination of mitochondrial inhibitors or mitochondrial biogenesis blockers with conventional chemotherapies would provide the desirable treatment for stubborn tumors.

Besides, mitochondrial dynamics was reported to be implicated in tumor malignant transformation and therapeutic resistance. The inhibition of mitochondrial fragmentation efficiently blocked the release of cytochrome c and the relevant cell death. Lung adenocarcinoma cells with an overexpression of OPA1 was positively correlated to cisplatin resistance while OPA1 downregulation induced the mitochondrial cristae deformation, thus increasing the release of cytochrome c and triggering apoptosis (25, 26). These studies demonstrated downregulation of OPA1 could partially reverse mitochondrial dynamics related cisplatin resistance. Santin et al. also reported that OPA1-mediated mitochondrial fusion was non-negligible reason for cisplatin resistance in neuroblastoma B50 rat cells (23). Similarly, MFN1/2 upregulated in L1210 cells was responsible for the resistance to cisplatin treatment, whereas the inhibition of mitochondrial fusion by silencing of MFNs sensitized neuroblastoma cells to cisplatin (24, 96). These studies suggested that targeting mitochondrial fusion might be a beneficial attempt to overcome chemotherapeutic resistance, as shown in Figure 5.

### Enhancement of Mitochondrial Plasticity Endows T Cells With the Robust and Persistent Immune Function

The metabolic competition between tumor cells and TILs for available glucose imposes nutrient deprivation on T cells and limits their ability to produce effector cytokines. As such, TILs are easy to display exhausted phenotype especially under immune-suppressive TME, which allows tumor evasion of immune recognition and eradication (66, 97, 98).

Rearming TILs with overexpression of mitochondrial biogenesis related genes contributed to persistent and efficient anti-tumor immunity (68, 69, 99). Improvement of mitochondrial biogenesis potentiated T cells with sufficient mitochondrial mass and SRC, which allowed for the production of durable and robust immune function even under immunosuppressive TME (73). Bengsch et al. reported that PD-1 signals suppressed the expression of PGC1α to inhibit mitochondrial biogenesis in CD8+ T cells, leading to the declined effector function in exhausted T cells (68). Conversely, PGC1α overexpression in hyporesponsive T cells was able to rescue their effector functions due to improvement of mitochondrial biogenesis. Consequently, identification of PGC1α stimulators could be an effective method for the development of mitochondrial therapies to resist TILs exhaustion.

**FIGURE 6:**
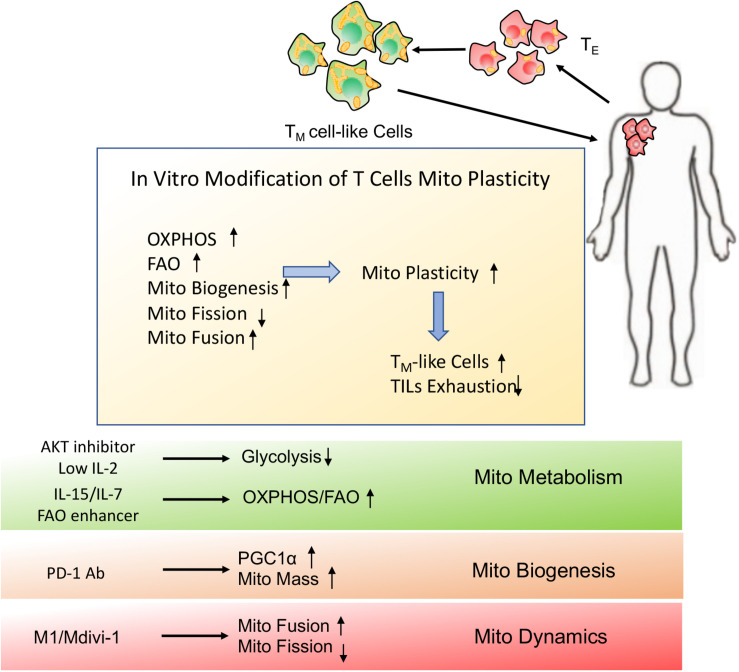
Schematic presentation of *in vitro* modification of T cells mitochondrial plasticity. *In vitro* modification of T cells mitochondrial plasticity through optimization of adoptive cellular immunotherapy (ACI) may beneficially facilitate T_M_ cell-like cells generation and prevent TILs exhaustion, which would better support T cells immune function and longevity even in the immunosuppressive microenvironment.

The success of immune response partially also relies on the long-lived T_M_ cells generation that can rapidly respond to antigen re-encounter. Manipulating T cells mitochondrial plasticity was used to enhance T cells long-term and persistent function through facilitation of the formation of T_M_ cell-like cells. First, increased glycolysis in T cells induced terminal differentiation, while decreased glycolysis contributed to more stable T_M_ cell-like cells generation (61, 100). For example, limiting glycolysis through culturing in low IL-2 media effectively retarded T cells terminal differentiation while promoting mitochondrial metabolism with IL-15 or IL-7 improved T cells effector function and longevity (101, 102). Second, manipulation of T cells mitochondrial plasticity with the promotion of mitochondrial biogenesis would better support their durable anti-tumor function. Inhibition of PD-1 activity has been attempted to enhance PGC1α mediated mitochondrial biogenesis, thus improving T_M_ cell-like immune response (68). Additionally, modification of mitochondrial morphology helped T cells switch to long-lived T_M_ cells, which optimally adapts to metabolic perturbations in the TME and rapidly responds to tumor re-emergence (17). Especially for adoptive cellular immunotherapy (ACI), modification of the patient’s anti-tumor T cells through mitochondrial morphology regulators may be a viable strategy to promote antitumor immunity. Culturing T cells with the “fusion promoter” M1 and the “fission inhibitor” Mdivi-1 contributes to T cells mitochondrial fusion, rendering them morphologically similar to T_M_ cells, as shown in Figure 6.

Together, promotion of mitochondrial metabolism, mitochondrial biogenesis and mitochondrial fusion endows T cells with the robust and persistent anti-tumor immune function. Therefore, development of therapeutic strategies to enhance the mitochondrial plasticity of T cells would offer a promising possibility to dramatically increase their immunotherapy efficacy.

## Conclusion

In summary, mitochondrial plasticity is controlled by mitochondrial dynamics, metabolic flexibility and mitochondrial biogenesis, which not only is required for tumor metastasis and therapeutic resistance but also endows T cells with durable and robust immune function. In this regard, metabolism-based therapies focusing on mitochondrial plasticity pinpoint the Achilles’ heel of tumor.

Given that tumor and T cells share the remarkably metabolic similarities, manipulating mitochondrial plasticity is required to strengthen the metabolic fitness of “friend” T cells but potentiate the metabolic vulnerabilities of “foe” tumor cells. Specifically, *in vivo* interference of tumor mitochondrial adaption through suppression of OXPHOS, mitochondrial fusion and mitochondrial biogenesis may dramatically inhibit tumor malignant progression and therapeutic resistance. Moreover, the combination of mitochondrial inhibitors or mitochondrial biogenesis blockers with conventional chemotherapies would provide more desirable treatment for stubborn tumors. Of course, strategies for delivering drugs through transporter-mediated drug uptake or nanoparticle-mediated delivery system would be used to improve the accuracy of drug administration. On the other hand, *in vitro* manipulation of TILs metabolic reprogramming through optimizing ACI or engineering chimeric antigen receptor T-cell (CAR-T) therapy may promote mitochondrial plasticity to beneficially facilitate T_M_ cell-like cells generation and prevent TILs exhaustion, which would optimally improve the efficacy of immunotherapy.

## Author Contributions

JZ provided the direction of this manuscript. HT designed and drafted this manuscript. LL and GW prepared the materials for this manuscript. BZ and HL were responsible for the figures of this manuscript. All authors contributed to the article and approved the submitted version.

## Conflict of Interest

The authors declare that the research was conducted in the absence of any commercial or financial relationships that could be construed as a potential conflict of interest.
